# Fabrication of *Antheraea pernyi* Silk Fibroin-Based Thermoresponsive Hydrogel Nanofibers for Colon Cancer Cell Culture

**DOI:** 10.3390/polym14010108

**Published:** 2021-12-29

**Authors:** Bo-Xiang Wang, Jia Li, De-Hong Cheng, Yan-Hua Lu, Li Liu

**Affiliations:** 1 School of Materials Science and Engineering, Shanghai University, Shanghai 200444, China; bxwang0411@163.com; 2 Liaoning Provincial Key Laboratory of Functional Textile Materials, Eastern Liaoning University, Dandong 118003, China; lj18840597623@163.com (J.L.); chengdehongldxy1@163.com (D.-H.C.); 3 School of Chemical Engineering, Eastern Liaoning University, Dandong 118003, China

**Keywords:** *Antheraea pernyi* silk fibroin, nanofibers, thermoresponsive, cancer cell, in vitro culture

## Abstract

*Antheraea pernyi* silk fibroin (ASF)-based nanofibers have wide potential for biomaterial applications due to superior biocompatibility. It is not clear whether the ASF-based nanofibers scaffold can be used as an in vitro cancer cell culture platform. In the current study, we fabricated novel ASF-based thermoresponsive hydrogel nanofibers by aqueous electrospinning for colon cancer (LoVo) cells culture. ASF was reacted with allyl glycidyl ether (AGE) for the preparation of allyl silk fibroin (ASF-AGE), which provided the possibility of copolymerization with allyl monomer. The investigation of ASF-AGE structure by ^1^H NMR revealed that reactive allyl groups were successfully linked with ASF. ASF-based thermoresponsive hydrogel nanofibers (p (ASF-AGE-NIPAAm)) were successfully manufactured by aqueous electrospinning with the polymerization of ASF and N-isopropylacrylamide (NIPAAm). The p (ASF-AGE-NIPAAm) spinning solution showed good spinnability with the increase of polymerization time, and uniform nanofibers were formed at the polymerization time of 360 min. The obtained hydrogel nanofibers exhibited good thermoresponsive that the LCST was similar with PNIPAAm at about 32 °C, and good degradability in protease XIV PBS solution. In addition, the cytocompatibility of colon cancer (LoVo) cells cultured in hydrogel nanofibers was assessed. It was demonstrated that LoVo cells grown on hydrogel nanofibers showed improved cell adhesion, proliferation, and viability than those on hydrogel. The results suggest that the p (ASF-AGE-NIPAAm) hydrogel nanofibers have potential application in LoVo cells culture in vitro. This study demonstrates the feasibility of fabricating ASF-based nanofibers to culture LoVo cancer cells that can potentially be used as an in vitro cancer cell culture platform.

## 1. Introduction

Cell culturing is a fundamental and essential process of biological sciences, for a wide range of basic and clinical in vitro research, which has led to outstanding developments in the fields of life science, biomedical, and biomaterial science [1]. The cell culture substrate requires suitable support materials with good biocompatibility, cell adhesion, growth and that are simple to produce. At present, cancer has become one of the major leading causes of death which affects millions of people worldwide [2]. The development of effective treatments for malignant tumors has been slowed by a lack of reliable in vitro culture models for researching the basic biological processes of the disease. Hence, investigation on formation, proliferation, invasion and metastasis are important research topics of malignant tumor. In this condition, the fabrication of in vitro cancer cell culture models (analogous to scaffolds in tissue engineering) that mimic the microenvironment where the cancerous cells reside and the tumor develops are in high demand [3]. Hydrogels have been investigated as a reliable material for cell culture and tissue engineering due to their high water content that can mimic the extracellular matrix (ECM) and regulate cell function [4,5,6,7]. Therefore, the hydrogel scaffold plays a pivotal role in supporting cells for culture as well as in tissue engineering. Scaffolds based on nanofibers were revealed to be a prototypical and favorable matrix for cell culture, showing superior capacity in shaping cell morphology and guiding cell migration when compared to other types of scaffolds [8]. Scaffold based nano- and sub-microfibers have gained increasing importance in applications in medical research, such as in vitro cancer cell culture [9], and especially in oncology [10]. So far, electrospinning has been considered a simple, effective, versatile and promising technique for producing nanoscale fibers with diameters ranging from tens of nanometers to several micrometers [11,12]. Electrospun nanofibers have a high specific surface area, with fiber diameters in the range of 10 nm to microns which can mimic the native ECM to enhance cell migration and proliferation, and high functionality which can be used as a barrier to prevent cells migrating between two different tissues [13]. Electrospinning nanofibers are also believed to be an efficient fabrication of in vitro cancer cell culture models for enhancing cell proliferation and adhesion.

Poly (N-isopropylacrylamide) (PNIPAAm) is the most intensively and widely investigated thermoresponsive polymer that exhibits a volume phase-transition in response to temperature changes at a lower critical solution temperature (LCST) around 32 °C [14]. PNIPAAm has the sharpest phase transition of all thermoresponsive N-alkylacrylamide polymers, and has been often utilized in tissue engineering [15,16], thermally modulated drugs [17,18] and gene delivery systems [19,20]. The utilization of a thermoresponsive polymer in the development of cell culture platforms allowed the dynamic control of cell adhesion and detachment in a desired manner by changing the temperature for targeted purposes. Particularly in the field of tissue engineering and regenerative medicine, thermoresponsive PNIPAAm exposed immense potential, as it enabled the creation and harvest of biologically functional products called “cell sheets” through the control of the dynamic environment for cells upon a change in temperature [21]. Hence, it has important applications for thermoresponsive polymers as cancer cell culture platforms, such as fluorescence tumor imaging [22], drug delivery in tumor screening and anti-cancer treatment [23]. PNIPAAm-based scaffolds as in vitro tumor culture models have good application in the previous reported, which encapsulate cells in physically cross-linked viscoelastic scaffolds at body temperature and easily release the cells by cooling to room temperature [24].

Numerous natural materials have been used to construct in vitro cancer cell culture models such as gelatin [25], collagen [26], fibrin [27] and hyaluronic acid [28], of which have been proven effective applications in cancer pathogenesis and development, anti-cancer mechanism and drug screening. Silk fibroin (SF) is an excellent candidate natural polymer for biomedical applications which has been widely used as a substrate for tissue regeneration of skin [29,30], bone [31], blood vessels [32], nerves and other tissue cells [33,34]. Silk proteins (fibroin and sericin) produced by silkworms are broadly classified into two types, which are mulberry and non-mulberry. *Antheraea pernyi* is a wild non-mulberry silkworm species belonging to the *Saturniidae* family, which is commonly known as Chinese temperate (oak) tussah. *A. pernyi* silk fibroin (ASF) derived from natural *A. pernyi* silkworm mainly consists of the primary structure of alanine (43.07%), glycine (27.27%), serine (11.26%), tyrosine (5.26%), and aspartic acid (4.47%) [35,36]. ASF can be regenerated into diverse forms of biomaterials and applied in biomedical fields because it contains the tripeptide sequences (Arg-Gly-Asp) known as RGD, whereby the integrin binding motif promotes the cell attachment [37]. It is reported that ASF nanofibers were successfully produced by electrospinning silk fibroin aqueous solution that had uniform and regular nanofibers with a diameter of 422 nm [38]. Therefore, electrospinning is a convenient and efficient technique to produce ASF-based nanofibers. However, Li X. et al. reported that continuous and regular nanofibers cannot be formed at low concentrations of ASF aqueous which usually required a concentration of more than 28.6% [39]. Silk fibroin is a self-assembling structural protein in natural silkworm fibers which induced the formation of crystalline β-sheet structure networks. The previously reported silk fibroin in nature is produced in the posterior section of the silkworm gland and is stored in the middle section, and contains a high content of random coil and α-helix. During fiber spinning into air, shear forces and elongational flow-induced self-assembly result in a structural transition of silk fibroin into the β-sheet structure, leading to the formation of solid fibers [40]. In vitro, purified silk fibroin aqueous solutions undergo self-assembly into β-sheet structures and form hydrogels [41]. The sol-gel transition of purified ASF aqueous solution mainly depends on concentration, temperatures, pH, shearing and ion strength [42]. The steady state of fresh ASF solutions is eventually destroyed by hydrophobic interactions or by van der Waals forces changing these factors. Random coil and α-helix to β-sheet structural transitions were induced by the change of these factors during the process of gelation. More importantly, the sol-gel transition of ASF solution happens easily under high concentration [43]. The gelation speed of ASF solution has close connection with solution concentration [44], whereby the β-sheets assemble into a physically cross-linked gel network under high concentration (only 5 min for the 10 wt% at 60 °C) [45]. Gels are stabilized due to the formation of thermodynamically stable β-sheet. Hence, the process of ASF aqueous electrospinning at high concentration still has limitation. Besides, electrospinning of regenerated ASF nanofibers is usually performed with volatile organic solvents, such as 1,1,1,3,3,3-hexafluoro-2-propanol (HFIP) [46], trifluoroacetic acid [47] and formic acid [48] with strong irritant. In previous research, it was revealed that ASF showed a poor spinnability and the electrospun fibers presented a belt-like morphology with a wide diameter distribution by using HFIP as ASF solvent [49,50]. To avoid the use of harsh solvents, it has been proved that the spinnability of polymer solution can be greatly improved by adding PEO [51,52]. It is reported that a heat treatment is applied and optimized to remove PEO and convert the amorphous Vectran-PEO nanofibers, which is performed in an oven in air in the temperature range 250–300 °C, and the treatment duration was in the range 15–24 h [53]. It can be seen that a complicated energy and time consuming process to remove PEO after spinning is required. Jordan A. M. et al. [54] reported a two-stage distillation process was used to recover methanol and water used in composite solvation to remove PEO. The results indicated that solvated PEO was recovered at a fractional recovery of 94 ± 4% at ~100% purity. It can be seen that there are still small amounts of PEO that are difficult to remove. More often, it will be difficult to confirm the complete absence of residual PEO in nanofibers.

Many previous researches have demonstrated the application of ASF-based scaffolds for tissue engineering, but few reports dealing with the ASF-based scaffolds for in vitro cancer cell culture can be found in literature. In our previous studies, it has been confirmed that ASF-based hydrogel support normal cell adhesion and growth [55]. Hence, good cytocompatibility is an important fundamental parameter for in vitro cancer cell culture. For this study, we attempt to develop novel ASF-based thermo-responsive hydrogel nanofibers using aqueous electrospinning without any harsh organic solvent, and explore whether they can be used as an in vitro platform for cancer cell culture. ASF was used as a nucleophilic reagent which reacted with allyl glycidyl ether (AGE) and provided the possibility of copolymerization with NIPAAm. The structure of ASF-AGE was researched by ^1^H NMR. ASF-based thermoresponsive hydrogel nanofibers (p (ASF-AGE-NIPAAm)) were successfully manufactured by aqueous electrospinning at the polymerization time of 360 min. The morphological, thermosensitivity of p (ASF-AGE-NIPAAm) hydrogel nanofibers were investigated by scanning electron microscopy (SEM) and differential scanning calorimetry (DSC). And the degradability of p (ASF-AGE-NIPAAm) nanofibers was evaluated in vitro. The obtained hydrogel nanofibers showed good thermoresponsive that the LCST was similar with PNIPAAm at about 32 °C, and good degradability in protease XIV PBS solution. In particular, the growth and adhesion of colon cancer (LoVo) cells cultured with p (ASF-AGE-NIPAAm) hydrogel nanofibers in vitro were assessed. LoVo cells grown on hydrogel nanofibers showed improved cell adhesion, proliferation, and viability. Based on the results, we conclude that p (ASF-AGE-NIPAAm) hydrogel nanofibers are suitable for in vitro culture of the LoVo cells line which demonstrated improved cell proliferation and viability.

## 2. Materials and Methods

### 2.1. Materials

Raw silk cocoons of *Antheraea pernyi* (Liaoning Tussah Silk Institute Co., Ltd., Dandong, China) were used for preparation of regenerated ASF. Ally glycidyl ether (AGE) was purchased from Sigma-Aldrich, St. Louis, MO, USA. N-isopropylacrylamide (NIPAAm) (98%, Aladdin Bio-Chem Technology Co., Ltd., Shanghai, China) was purified by dissolution and recrystallization in hexane. All other materials and reagents were purchased from Sinopharm Chemical Reagent Co., Ltd. Shanghai, China, and used as supplied.

### 2.2. Preparation of Allyl Silk Fibroin (ASF-AGE)

**Figure 1:**
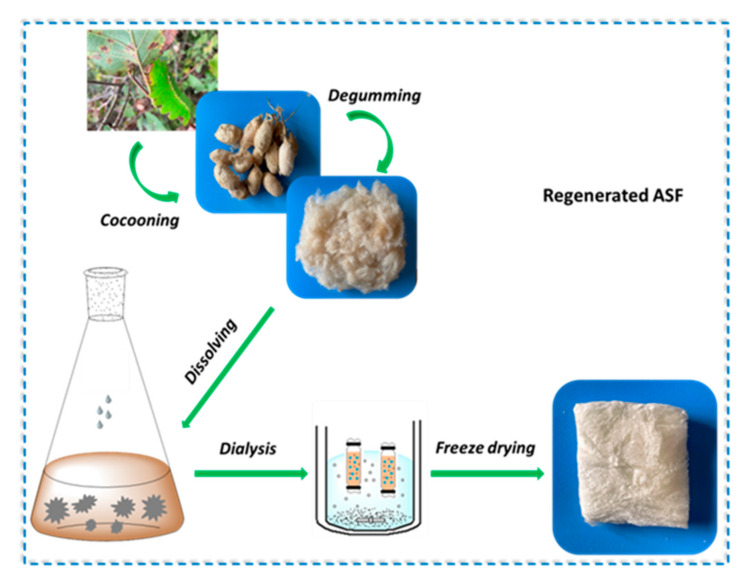
Preparation of regenerated *A. pernyi* silk fibroin.

Raw cocoons of *A. pernyi* were degummed by boiling for 30 min in an aqueous solution of 0.5% Na_2_CO_3_ three times, and then rinsed thoroughly with deionized water. The degummed silk was dried at 40 °C for two days prior to use. After drying, the degummed fibers were dissolved in molten Ca(NO_3_)_2_ at 105 °C for 4 h. The dissolution process was kept under stirring to guarantee the completely dissolution. The mixed solution was dialyzed against deionized water for 3 days with an 8–14 kDa molecular weight cut-off dialysis tube to remove salts. The solution was finally centrifuged at 8000 rpm for 10 min to remove impurities and frozen at −50 °C for 12 h, followed by freeze-drying for 48 h to obtain regenerated ASF sponge (Figure 1).

The regenerated ASF sponge sample was accurately weighted by an electronic balance (ME103E, METTLER TOLEDO, Zurich, Switzerland) and padded into a 250 mL three-necked round-bottomed flask with distilled water dissolved to a concentration of 10 mg/mL. The ASF solution was treated by 2 M Na_2_S_2_O_3_ catalyst solution to adjust the pH to 10 ± 0.2, which catalyzed the nucleophile (ASF) to attack the carbon center atom of the epoxy group under this alkaline condition. Then nitrogen, as a protective gas (isolate the air), was continuously injected into the flask together with AGE that was dripped into it slowly. This flask was joined with a reflux condenser and reacted in a thermostatically controlled bath with stirring at the desired temperature. At the end of reaction, the mix solution was treated by 1 M HCl solution to adjust the pH to neutral. The solution was dialyzed against deionized water for 3 days with an 8–14 kDa molecular weight cut-off dialysis tube (diameter 28 mm, flatten width 44 mm, Viskase, Chicago, IL, USA) at 4 °C, the solution was rapidly frozen at −50 °C for 8 h, then the allyl silk fibroin (ASF-AGE) was obtained by freeze-drying.

### 2.3. Preparation of ASF-Based Thermoresponsive Hydrogel Nanofibers (p (ASF-AGE-NIPAAm))

NIPAAm was dissolved in a mixture of toluene and n-hexane (6:4, v/v) solution and a concentration of 15 wt% of NIPAAm solution was prepared. The NIPAAm solution was heated to 60 °C in a thermostatically controlled bath and cooled at room temperature, then placed in a −5 °C fridge until crystal was completely precipitated and the purified NIPAAm was obtained. The purification of NIPAAm was for the sake of removing the inhibitor (MEHQ). ASF-AGE was dissolved in deionized water to a concentration of 5%, then mixing with APS and TEMED in an ice bath. NIPAAm solution (75 mg/mL) was slowly dripped into the mixed solution with nitrogen injected continuously at 20 °C. The formulation of p (ASF-AGE-NIPAAm) spinning solutions was listed in Table 1. After a period of polymerization, the sticky solution (poor liquidity and sticky feeling) was obtained, and the surface tension of p (ASF-AGE-NIPAAm) solution was tested by a surface tension meter (QBZY-2 0–400 mN/m, Shanghai, China) at 15 and 30 °C. The viscosity of p (ASF-AGE-NIPAAm) solution was measured with a rotational rheometer (RheoStress 1, HAAKE, Karlsruhe, Germany) with a 25 mm cone plate at 15 and 30 °C.

The primary p (ASF-AGE-NIPAAm) spinning solutions were loaded into a 10 mL syringe equipped with a 0.5 mm diameter stainless steel needle. The electrostatic spinning machine (FM-11, Beijing, China) was purchased from Beijing Future Material Sci-tech Co., Ltd. which had a high voltage direct current (HVDC, 0~50 kV) with a roller collector (100–2000 rpm). The syringe pump (JNS-02, Nanjing, China, 0.1 µL/h~681.73 mL/min), injector (0.5~200 mL) and stainless steel needle were purchased from Janus New-Materials Co., Ltd. Then, the p (ASF-AGE-NIPAAm) hydrogel nanofibers were produced by the electrostatic spinning system at a stepping rate of 1.5 µL/s under 20 kV voltage field and 12 cm distance between tip and receptor for 15 min at 24 °C and 60% relative humidity. Finally, spun electrospun p (ASF-AGE-NIPAAm) nanofibers were treated with water vapor and immersed in 75% ethanol aqueous to insolubilize it by inducing β-sheet structure formation.

### 2.4. ^1^H Nuclear Magnetic Resonance (^1^H-NMR)

The 5 mg products of ASF and ASF-AGE were dissolved in 1 mL of deuterium oxide. The ^1^H-NMR spectra of each sample were recorded on a Bruker AV400 NMR spectrometer (Bruker, Karlsruhe, Germany) with an operating frequency at 400 MHz.

### 2.5. Differential Scanning Calorimetry (DSC)

PNIPAAm is a smart synthetic polymer that presents a hydrophilic-hydrophobic thermoresponsive phase transition around 32 °C, known as its lower critical solution temperature (LCST). The DSC technique was employed in order to obtain thermal (endothermic or exothermic) curves and determine the LCST for the sample [56]. The LCST of p (ASF-AGE-NIPAAm) hydrogel nanofibers sample was determined by using a differential scanning calorimeter (Q2000, TA instruments, New Castle, DE, USA) under nitrogen at a flow rate of 20 mL/min. The weight of samples was kept at about 10 mg. The above sample was put in the aluminum sample holder and frozen below −20 °C. The samples were performed in the temperature range 0~50 °C at a heating rate of 2 °C/min.

### 2.6. Morphology of p (ASF-AGE-NIPAAm) Hydrogel Nanofibers

The morphology of p (ASF-AGE-NIPAAm) hydrogel nanofibers were observed using a scanning electron microscope (JSM-IT100, JEOL, Tokyo, Japan) along with sputtering a thin layer of gold at 20 kV.

### 2.7. Contact Angle Measurements

Static water contact angle measurements were accomplished by using a drop shape analyzer (PT-705, Zhongchen Digital Technology, Shanghai, China) with a needle method at a flow rate of 1.0 µL/s in 36% relative humidity, and the test temperatures were 25 and 45 °C, respectively. The Young Laplace drop profile fitting method was applied to evaluate the static contact angle results. The needle specifications were as follows: Diameter—0.5 mm and the water droplet volume—~5 µL. The standard deviation of the measurement series might create the error bars. The measurement was repeated three times.

### 2.8. Degradability of p (ASF-AGE-NIPAAm) Hydrogel Nanofibers

The p (ASF-AGE-NIPAAm) hydrogel nanofibers sample (Nano-6) was cut in a 1 ± 0.2 cm × 1 ± 0.2 cm film. Then, the sample was weighted and incubated in the same volume (30 mL) of PBS solution (pH = 7.4) and 5.0 U/mL protease XIV PBS solution (bath ratio 1:80), respectively. The sample was placed in a test tube in a constant temperature incubator (HZQ-F160A, Yiheng Scientific Instrument, Shanghai, China) at 37 °C for 14 days under slow shaking. At regular intervals, the sample was lyophilized and weighed and each sample was evaluated in triplicate. The measurement was repeated three times for final results. Degradation rate was expressed as the percentage of weight loss relative to the initial dry weight. The weight loss ratio (WLR) was calculated using Equation (1):

(1) $$\mathrm{WLR}\left(\%\right)=\frac{W_{o}-W_{t}}{W_{o}}\times 100$$

where $W_{o}$ and $W_{t}$ are the weights of nanofibers sample before and after degradation, respectively.

### 2.9. Cell Culture

Colon cancer (LoVo) cells (frozen in −80 °C ultra-cold storage freezer) were cultured in 4 mL of Dulbecco’s modified Eagles medium (DMEM, Meilun-bio, Dalian, China) which was mixed with 10% fetal bovine serum (FBS, HyClone, Logan, UT, USA), 100 U/mL penicillin and 100 μg/mL streptomycin, at 37 °C in an atmosphere of 5% CO_2_ in air. In the logarithmic growth phase, the cells were trypsinized using 0.25% trypsin (Invitrogen, Waltham, MA, USA) and resuspended at 1.0 × 10^5^ cells/mL in fresh DMEM with FBS and antibiotics. A 100 μL aliquot of cells suspension was inoculated in each well of the 96-well cell culture plates to cover the p (ASF-AGE-NIPAAm) nanofibers (*n* = 6); a 100 μL aliquot of cells without samples was the control group and the blank group was not inoculated with cells. The 96-well cell culture plates were kept in a humidified atmosphere of 95% air and 5% CO_2_ at 37 °C for 1, 3, 5 and 7 days. After culturing, a portion of cells were washed three times with DMEM. The medium was replaced with 100 μL DMEM and mixed with 10 μL MTT agent for 4 h for the cell viability test. After incubation, the reaction was carefully removed from each well, and 100 μL dimethylsulfoxide (DMSO) was added. The plates were gently agitated until the blue-purple crystal (formazan precipitate) completely dissolved, and the absorbance at 490 nm was measured using a Synergy H1 microplate reader (Bio-Tek, Winooski, VT, USA). The other cells were dipped into 4% paraformaldehyde solution for 15 min, and washed with PBS solution three times. These cell samples were blocked with PBS containing 1% Triton X-100 by staining with DAPI (Sigma, St. Louis, MO, USA). After incubation in the dark for 40 min at room temperature, a confocal laser scanning microscope (CLSM) (FV-1200, Olympus, Tokyo, Japan) was used to observe the cells adhesion and growth to the sample.

## 3. Results and Discussion

### 3.1. Synthesis Allyl Silk Fibroin (ASF-AGE)

**Figure 2:**
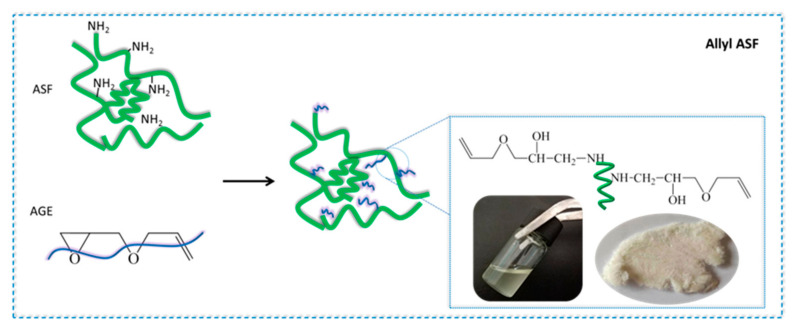
A schematic illustration of the synthesis of ASF-AGE.

The reaction of epoxides with protein fibers, such as silk and wool, has been extensively researched [57,58,59]. Hence, silk fibroin can be chemical modified with an epoxy group by its reactive groups of amino, hydroxyl or carboxyl. The ASF was dissolved in deionized water, and the ASF solution was adjusted to 10 mg/mL. The ASF solution was treated by 2 M Na_2_S_2_O_3_ catalyst solution to adjust the pH to 10 ± 0.2. In this condition, the AGE solution was gradually dripped into it; the nucleophilic substitution occurred with the amino group in ASF and the epoxy group in AGE. The aggregated structure of ASF in solution shows a random coil structure, and numerous terminal amino groups are exposed. A nucleophilic substitution reaction occurred by amine groups of ASF with epoxide groups of the diglycidyl ether (Figure 2). A base-catalyzed ring opening of the epoxide groups occurs predominantly at the least hindered carbon atom [60].

The use of affinity techniques has become a routine laboratory method in biochemical research. As is well known, epoxides react with amines, alcohols, phenols, thiols and carboxylic acids [61]. The main peptide chains of proteins carry side chains of all these types and the earliest study of the reaction with epoxy compounds dates from 1944 [62]. Therefore, the reaction of epoxides with silk fibroin is still a subject of great interest for both fundamental and applied reasons. Recently, some researches have reported silk fibroin that was chemically modified and cross-linked with epoxides by its reactive groups of amino, hydroxyl or carboxyl [57,58].

**Figure 3:**
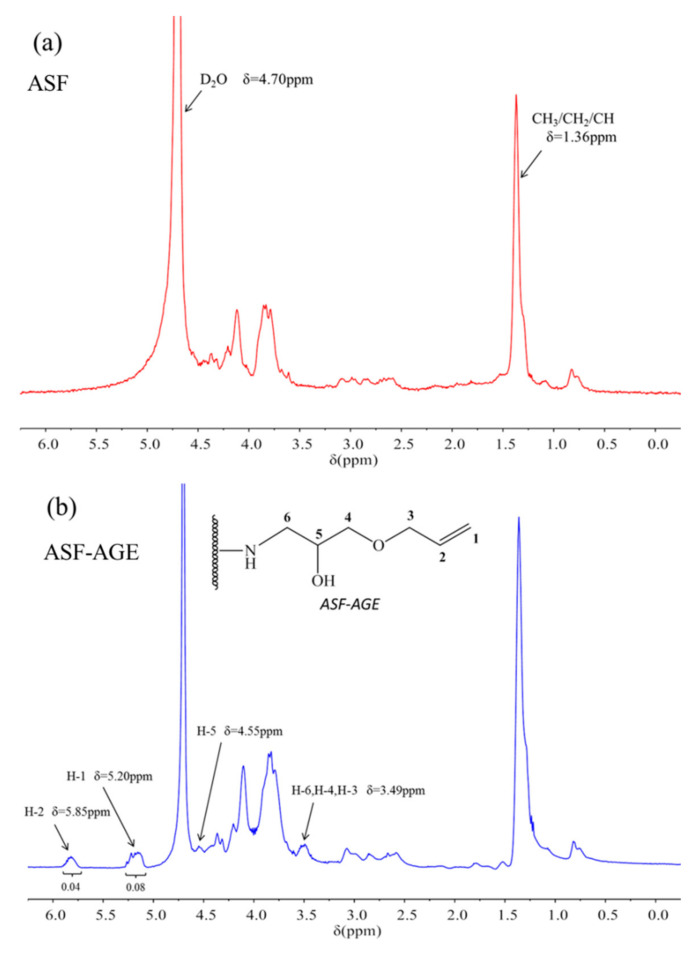
^1^H NMR spectra of (**a**) ASF and (**b**) ASF-AGE.

In the previous literature [63], Dmitrieva L. L. et al. studied the cyclization of 1-allyloxy- and 1-[2-(vinyloxy) ethoxy]-3-(2-propynyloxy)propan-2-ols in water, and reported that the ^1^H NMR spectra characteristic signals from protons in the allyloxy group were δ = 5.26 ppm d.d.t (CH_2_=) and δ = 5.89 ppm d.d.t (CH=). In this study, for the ^1^H NMR spectra of ASF and ASF-AGE (shown in Figure 3), the characteristic signals of ASF-AGE (Figure 3b) appeared at 5.20 ppm (H-1) and 5.85 ppm (H-2), which were mainly attributed to the characteristic signals of protons resonances on the allyloxy group, and coincided with the results of literature. Meanwhile, the strength of protons resonances on the allyloxy group (CH_2_= and CH=) was 2:1. This result has also been confirmed in literature which matches with the ^1^H NMR spectra for AGE [64]. The chemical shift at 4.55 ppm in Figure 3b was attributed to the characteristic signals of the ring-opening epoxy group (H-5), and 3.49 ppm corresponded to the methylene (-CH_2_-) protons (H-6, H-4, H-3) resonances, which coincided with the chemical shifts of the AGE in a previous report [65]. Whereas there were no corresponding chemical shift characteristic signals in the spectra of ASF (Figure 3a). It was reported that glycidyl ether can be easily reacted with the silk fibroin in the presence of the salt [66]. Meanwhile, it has been reported that, to produce a secondary amine and a hydroxyl group, the reaction is between an epoxide ring and a primary amine [67]. Hence, according to the ^1^H NMR spectrum, it was found that nucleophilic substitution occurred with the ASF amino group and the AGE epoxy group, and reactive allyl double bonds were obtained on ASF.

### 3.2. Fabrication of p (ASF-AGE-NIPAAm) Hydrogel Nanofibers

**Figure 4:**
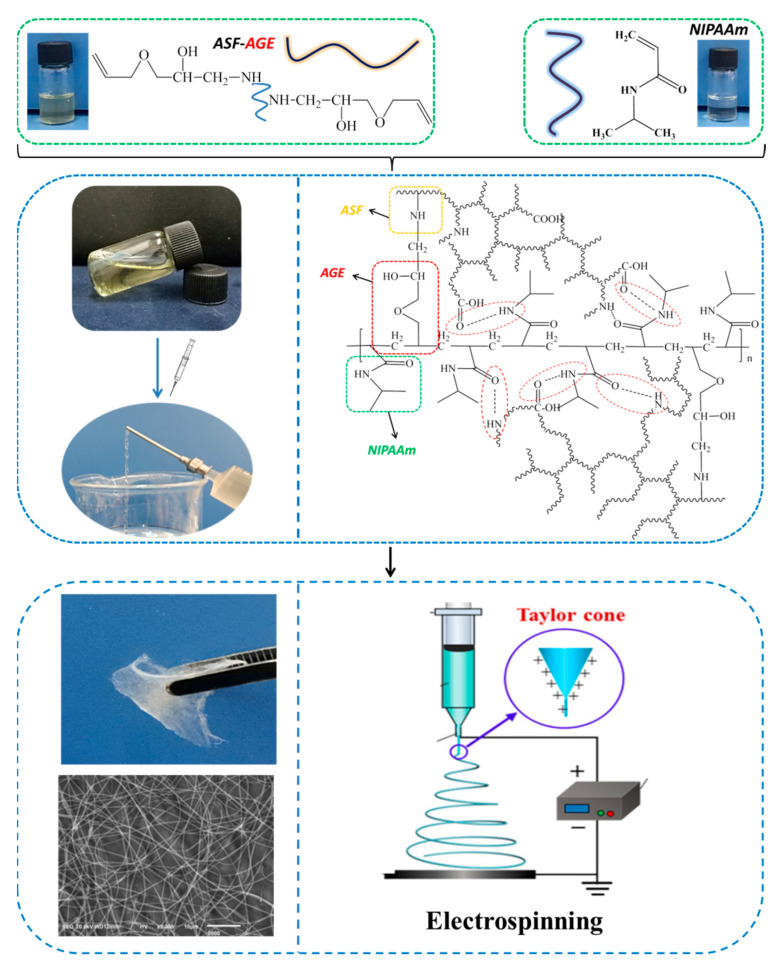
Preparation of p (ASF-AGE-NIPAAm) hydrogel nanofibers.

At present, most electrospun ASF-based nanofibers are fabricated from organic solvents, such as HFIP, TFA and formic acid. One expected application of the electrospun SF non-woven mats is for cellular scaffolds in regenerative medicine [68,69]. For medical use, the components in materials production, including raw material, solvent and reagent, must be safe for cells and living human bodies [70]. However, most organic solvents may cause potential toxicity towards the encapsulated cells, and residual organic solvents will definitely cause damage to cells and human bodies. In this study, ASF-AGE can be copolymerized with allyl monomer with the condition of initiator, and spinnability was improved with the increase in viscosity of the polymerization. The fabrication of p (ASF-AGE-NIPAAm) nanofibers is shown in Figure 4. Firstly, an amount of SO^4−^ radicals were decomposed by APS under the catalysis of TEMED, which attacked the allyl C=C of ASF-AGE and NIPAAm, and copolymerization occurred between ASF-AGE and NIPAAm. Then, it was covalently connected with the allyl C=C in ASF-AGE together with the formation of the PNIPAAm polymer long chain. Finally, the solution system gradually became sticky, with the formation of a hydrogen bond between ASF peptide chains and PNIPAAm, with the extension of polymerization time.

**Figure 5:**
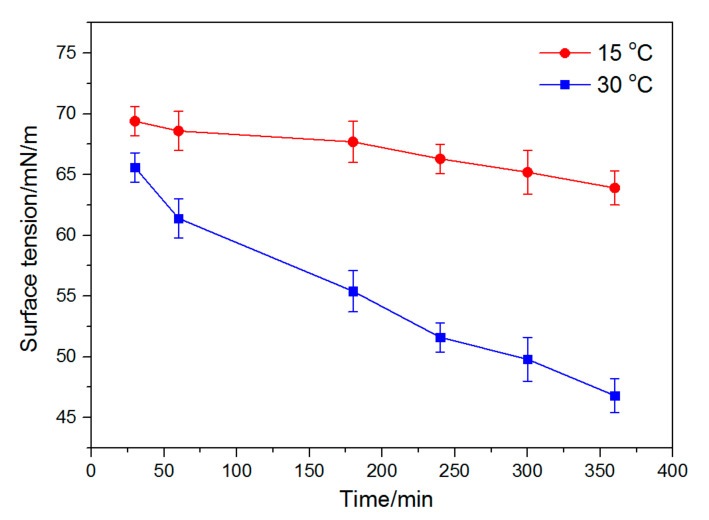
Surface tension of p (ASF-AGE-NIPAAm) spinning solution with different polymerization times at 15 and 30 °C.

**Figure 6:**
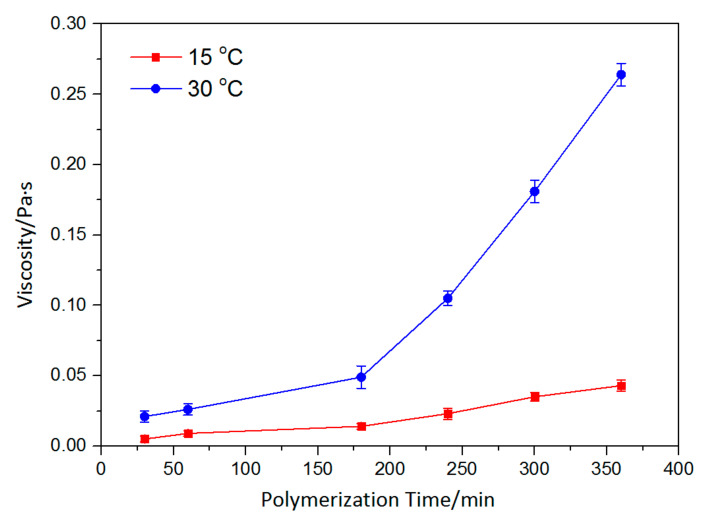
Viscosity of p (ASF-AGE-NIPAAm) spinning solution with different polymerization times at 15 and 30 °C.

To demonstrate the spinnability of p (ASF-AGE-NIPAAm) solution, p (ASF-AGE-NIPAAm) spinning solution with different polymerization times was researched. Polymerization time determined the spinnability of the solution through effects on solution viscosity and surface tension. The surface tension and viscosity of spinning solution is shown in Figure 5 and Figure 6, respectively. It was shown that the surface tension of spinning solution at 30 °C was decreased faster than that at 15 °C. The viscosity of the spinning solution had a great change at 30 °C. It was reported that surface tension is mainly influenced by the interaction between the polymer and solvent [71]. Meanwhile, the surface tension and viscosity are altered as the concentration of polymer is increased [72]. According to that reported, we can believe that it due to effective polymerization occurring at a higher temperature, which increased the concentration of polymer in spinning solution. The viscosity of p (ASF-AGE-NIPAAm) spinning solution gradually increased with polymerization time at 30 °C. In particular, the variation in the viscosity was dramatically increased when the polymerization time exceeded 300 min, implying increased concentration of p (ASF-AGE-NIPAAm) macromolecules and strong interaction with aqueous solution. 

**Figure 7:**
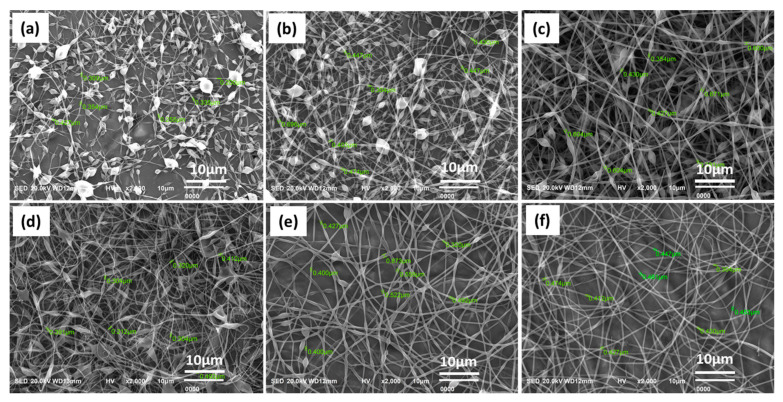
The SEM images of p (ASF-AGE-NIPAAm) hydrogel nanofibers with different polymerization times: (**a**) 30 min, (**b**) 60 min, (**c**) 180 min, (**d**) 240 min, (**e**) 300 min and (**f**) 360 min.

The SEM images of p (ASF-AGE-NIPAAm) hydrogel nanofibers with different polymerization times at 30 °C are shown in Figure 7. It was shown that p (ASF-AGE-NIPAAm) hydrogel nanofibers were not formed in a short polymerization time (30~180 min) (Figure 7). Discontinuous nanofibers with numerous undifferentiated drops are shown in Figure 7a–c with average diameters of 311 ± 115 nm (Figure 7a), 417 ± 113 nm (Figure 7b), and 508 ± 155 nm (Figure 7c), respectively. This is because the viscosity of spinning solutions was not high enough to form a stable electrospinning jet. There were a large number of liquid beads electrosprayed on the collector surface and continuous nanofibers cannot form under a short time period (Figure 7a–c). Fong et al. [73] reported that when the polymer has a higher molecular weight, a fiber between the beads is created to make the “beads-on-a-string”. This is caused by the contraction of the jet due to surface tension. As the viscosity of the spinning solution is increased, and subsequently the number of chain entanglements increases, the beads become bigger and more spaced out. Eventually a beadless fiber is formed. According to this, it follows that the insufficient polymerization time of p (ASF-AGE-NIPAAm) results failed to form enough molecular weight. The polymer concentration is low in solution. Therefore, the beads structure in the fiber tends to form for polymer solution at low concentration. Increased polymerization from 180 to 300 min significantly decreased the number of beads in nanofibers, with average diameters of 370 ± 155 nm (Figure 7d) and 466 ± 95 nm (Figure 7e). Uniform and regular p (ASF-AGE-NIPAAm) hydrogel nanofibers with a diameter of 452 ± 120 nm were obtained when the polymerization time was extended to 360 min (Figure 6f). The results demonstrated good spinnability with the extension of polymerization time, and uniform hydrogel nanofibers were formed at the polymerization time of 360 min.

### 3.3. Thermoresponsive of p (ASF-AGE-NIPAAm) Hydrogel Nanofibers

**Figure 8:**
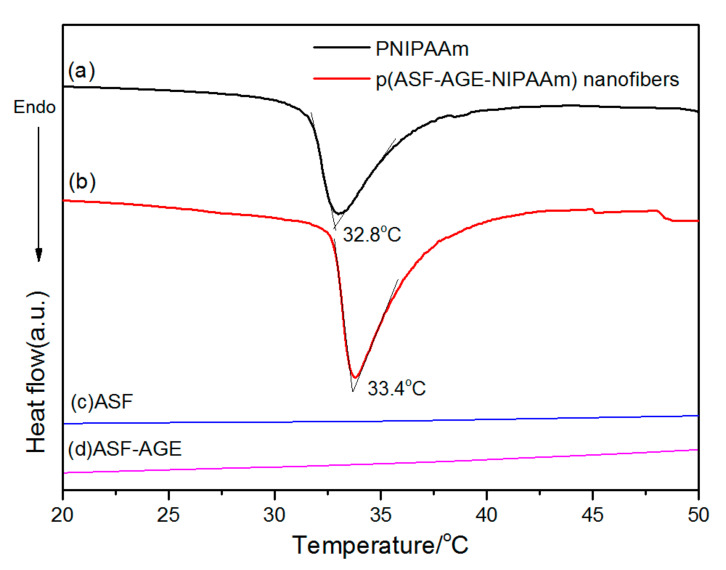
DSC curves for (**a**) PNIPAAm, (**b**) p (ASF-AGE-NIPAAm) hydrogel nanofibers, (**c**) ASF and (**d**) ASF-AGE.

The thermoresponsive of the p (ASF-AGE-NIPAAm) hydrogel nanofibers sample (Nano-6) was examined by DSC (Figure 8). The onset temperature of the endotherm was referred to as the low critical solution temperature (LCST). Obviously, the p (ASF-AGE-NIPAAm) hydrogel nanofibers showed almost the similar LCST at around 32 °C, a very useful temperature for biomedical applications since it is close to the body temperature, which matched well with the LCST of PNIPAAm. Hydrogen bonds between water and hydrophilic groups make the hydrogels swell when the temperature is below the LCST. Once the external temperature increases above the LCST, the hydrophobic interaction among hydrophobic groups dominates, resulting in a phase separation and shrinkage of hydrogels [74]. PNIPAAm is one typical polymer which shrinks in high temperature. A polymer solution below the LCST is a clear, homogeneous solution, while a polymer solution above the LCST appears cloudy [75]. In the present study, ASF consisted of a large amount of alanine and glycine, which are super hydrophobic. However, the hydrophilicity/hydrophobicity structure of PNIPAAm was not effectively changed. Thus, there was little impact on the LCST of PNIPAAm in the presence of ASF. The result indicated that p (ASF-AGE-NIPAAm) hydrogel nanofibers have a similar thermoresponsive to PNIPAAm.

### 3.4. Wettability of p (ASF-AGE-NIPAAm) Hydrogel Nanofibers

**Figure 9:**
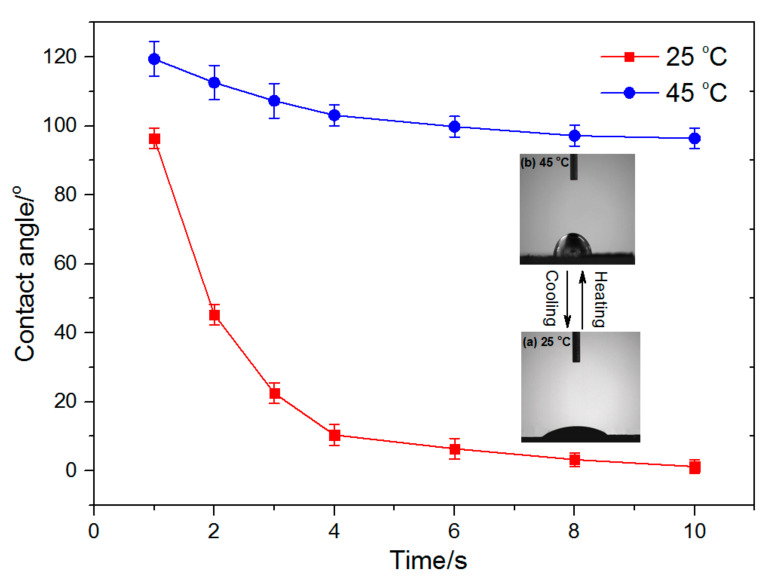
Time dependence of water contact angle for Nano-6 nanofibers sample at different temperatures (the insert images were the water droplet shape on the Nano-6 sample in 8 s at (**a**) 25 °C and (**b**) 45 °C).

The wettability of p (ASF-AGE-NIPAAm) hydrogel nanofibers (Nano-6) with temperature changes are shown in Figure 9. The water droplet shape on the surface of the Nano-6 sample at 25 and 45 °C are also shown in Figure 9. At 25 °C, the suspended water droplet was rapidly absorbed after 8 s and the contact angle was close to 0°. At this time, the p (ASF-AGE-NIPAAm) hydrogel nanofibers showed to be super hydrophilic. When the temperature increased to 45 °C, the suspended water droplet was not immersed into the nanofibers and the contact angle changed non-remarkably to 119.4 ± 5°and 96.4 ± 3° after 10 s (Figure 9). This behavior implied that wettability of p (ASF-AGE-NIPAAm) hydrogel nanofibers could switch from hydrophilicity to hydrophobicity when the temperature increased from 25 to 45 °C, which was consistent with the literature [76]. The results show that p (ASF-AGE-NIPAAm) hydrogel nanofibers are thermoresponsive. At temperatures lower than LCST, water molecules can make contact with hydrophilic PNIPAAm molecules. At temperatures higher than LCST, even combined with the ASF-AGE, the nanofibers still showed hydrophobicity. The result indicated that the PNIPAAm played an important role in the switch of wettability in the p (ASF-AGE-NIPAAm) hydrogel nanofibers. According to previous literature [77], it indicates the switchable wettability of PNIPAAm-based nanofibers can both support cell viability and permit cell sheet detachment, which provides possibility for further research in cell culture.

### 3.5. Degradation Behavior of p (ASF-AGE-NIPAAm) Hydrogel Nanofibers

**Figure 10:**
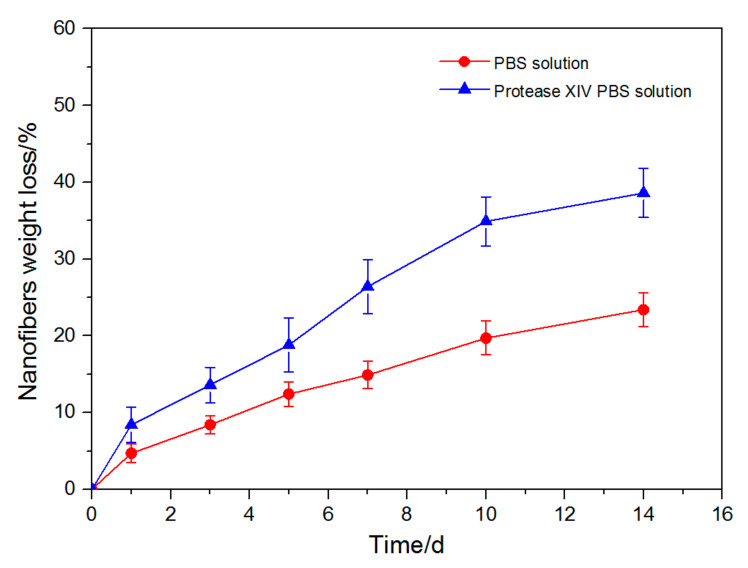
Degradation of p (ASF-AGE-NIPAAm) hydrogel nanofibers (Nano-6 sample) in the PBS and protease XIV PBS solution at 37 °C.

The development of a biodegradable nanofibers scaffold is nowadays of increasing interest and deserves the attention of academic and biomedical research. In this study, in vitro degradation of p (ASF-AGE-NIPAAm) hydrogel nanofibers (Nano-6 sample) was monitored, by examining the weight loss ratio with incubation time in the PBS solution and protease XIV PBS solution at 37 °C, respectively, as presented in Figure 10. As shown in Figure 10, the weight loss of the Nano-6 sample followed a clear trend after exposure to PBS and protease XIV PBS solution. During incubation with PBS buffer solution, the nanofibers absorb water with strong swelling stresses. With the increased strength of the swelling stresses, it becomes higher than that of hydrophobic interactions, which hold together the thermosensitive chains within hydrophobic domains and polymer dissociation occurs, eventually leading to disintegration [78]. Under the degradation condition without enzymes, hydrolysis of amide bonds dominates degradation. The result is consistent with the literature [79]. The weight loss of the Nano-6 sample was 23.4% after 14 days in PBS solution, whereas the Nano-6 sample showed increased weight loss of approximately 38.6% after 14 days in protease XIV PBS solution. After incubation for 14 days in protease XIV PBS solution, the weight loss ratio of nanofibers was higher than in PBS solution. During incubation with protease XIV PBS solution, the degradability of p (ASF-AGE-NIPAAm) hydrogel nanofibers was significantly enhanced. This result implied that the ASF component in nanofibers was degraded by enzymes, which is consistent with the previous report [80]. It has been reported that the silk fibroin can be degraded by proteinase in vitro and in vivo, and the degradation products of silk-based materials are soluble peptides and free amino acids [81]. In our previous study, the formation of p (ASF-AGE-NIPAAm) hydrogels had mainly a physical cross-linked structure without any chemical cross-linked components, and the aggregated structure of hydrogels mainly existed in the form of an amorphous and lower metastable β-sheet physical structure [55]. The amorphous regions and metastable silk I structure were preferentially degraded in regenerated silk fibroin materials [82]. Therefore, in vitro degradation results indicated that the p (ASF-AGE-NIPAAm) hydrogel nanofibers were more easily degraded in the protease XIV PBS solution.

### 3.6. Cell Adhesion and Growth on p (ASF-AGE-NIPAAm) Hydrogel Nanofibers

**Figure 11:**
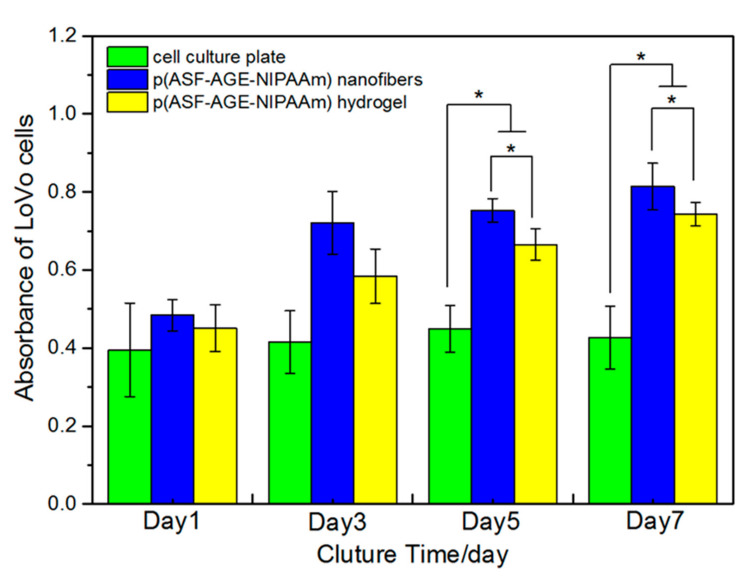
MTT assay of the proliferation of LoVo cells seeded onto different substrates after one, three, five and seven days of co-culture (* *p* < 0.05).

To show the effectiveness of cancer cell adhesion and growth on p (ASF-AGE-NIPAAm) hydrogel nanofibers, cell culture was performed on the Nano-6 sample with LoVo cells. The yellow MTT was reduced to a purple formazan dye by mitochondrial dehydrogenase in living cells, and can be used to assess cell viability [83]. After culture, the LoVo cells were stained with DAPI (blue) for the nucleus. The OD values of purple formazan at 490 nm are proportional to the number density of adhered cells. As shown in Figure 11, the OD values confirmed the presence of cells, suggesting a successful loading of cells that increased over culture time. The adherence of cell culture plate was lower than nanofibers in seven days (* *p* < 0.05). It was shown that more cells grew on p (ASF-AGE-NIPAAm) hydrogel nanofibers than on hydrogel (* *p* < 0.05). This was due to the difference in surface area between the nanofibers and normal hydrogel. The nanofibers sheet had a larger area and more space for cell adhesion and growth than the normal hydrogel because of its porosity [84].

**Figure 12:**
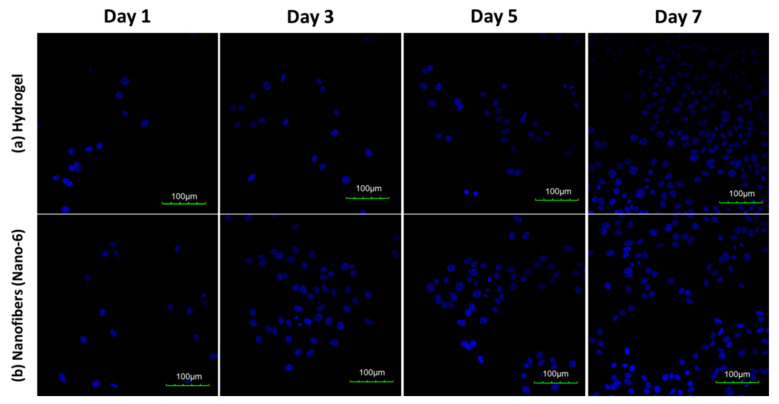
CLSM images of LoVo cells on (**a**) hydrogel and (**b**) hydrogel nanofibers (Nano-6 sample). The fluorescence is blue labeled by DAPI (a marker for the cell nucleus), and the scale bars are 100 μm. All samples were cultured for one, three, five and seven days.

The CLSM images of the LoVo cells cultured on different substrates for one, three, five and seven days are shown in Figure 12. At day one, few cells adhered on the p (ASF-AGE-NIPAAm) hydrogel nanofibers and hydrogel. The number of LoVo cells increased by several times by day three. The density number of LoVo cells was significantly increased at days five and seven. From day one to day seven, the cell density number and growth on p (ASF-AGE-NIPAAm) hydrogel nanofibers were higher than on hydrogel. Furthermore, the MTT assay also confirmed this result. The cell culture results suggested that it was beneficial for colon cell adhesion and growth, with the p (ASF-AGE-NIPAAm) hydrogel nanofibers as the LoVo cell culture substrate. In addition, the cell adhesion density of p (ASF-AGE-NIPAAm) hydrogel nanofibers was more obvious than for hydrogel.

## 4. Conclusions

Novel ASF-based thermoresponsive hydrogel nanofibers were successfully fabricated by aqueous electrospinning for colon cancer (LoVo) cells culture. The p (ASF-AGE-NIPAAm) spinning solution showed good spinnability with increased polymerization, and uniform nanofibers were formed with a diameter of 452 ± 120 nm at the polymerization time of 360 min. The hydrogel nanofibers revealed obvious thermoresponsive behavior, of which the LCST was about 32 °C. The p (ASF-AGE-NIPAAm) hydrogel nanofibers can be easily degraded in the protease XIV PBS solution. Furthermore, by culturing colon cancer (LoVo) cells in vitro, the LoVo cells grown on hydrogel nanofibers showed improved cell adhesion, proliferation, and viability than those on hydrogel. This is a convenient and feasible approach to fabricate ASF-based functional nanofibers in the application of cancer cell culture. We aim to present a valuable route for developing an ASF-based cancer cell culture platform.

## Figures and Tables

**Table 1:** The formulation of p (ASF-AGE-NIPAAm) spinning solutions.

Code	ASF-AGE (mg)	NIPAAm (mL)	APS (mg)	5% TEMED (µL)	Polymerization Time (min)	Conductivity (mS/m)
Nano-1	200	4.0	6.0	60	30	96.2 ± 4.2
Nano-2	200	4.0	6.0	60	60	94.1 ± 3.3
Nano-3	200	4.0	6.0	60	180	93.4 ± 2.7
Nano-4	200	4.0	6.0	60	240	94.5 ± 3.6
Nano-5	200	4.0	6.0	60	300	96.7 ± 2.4
Nano-6	200	4.0	6.0	60	360	97.1 ± 1.5

## Data Availability

Not applicable.
